# Antibacterial and Antifungal Activity and Acute Toxicity of Crude Extracts From the Wild Edible Mushrooms *Cantharellus veraecrucis*, *Cantharellus violaceovinosus*, and *Turbinellus floccosus*

**DOI:** 10.1155/ijfo/5004650

**Published:** 2025-04-15

**Authors:** Antero Ramos, Guillermo Mendoza, Leonardo Serrano-Márquez

**Affiliations:** ^1^Red de Biodiversidad y Sistemática, Instituto de Ecología, A.C., Xalapa, Veracruz, Mexico; ^2^Centro de Investigación en Micología Aplicada, Universidad Veracruzana, Xalapa, Veracruz, Mexico

## Abstract

Many phytopathogenic and human pathogenic microorganisms have increased their resistance to conventional antibiotics and fungicides, which is why the most recent research has focused on exploring new strategies for their management and control. One of these strategies is the search for new natural compounds present in wild edible macrofungi, which in early research have shown evidence of having bioactive properties and high nutritional value, profitability, and biodegradability, among other benefits. The present study is aimed at determining the antibacterial and antifungal activity and acute toxicity of crude extracts obtained from fruit bodies of the wild mushrooms *Cantharellus veraecrucis*, *Cantharellus violaceovinosus*, and *Turbinellus floccosus*. The results revealed that the three fungal species have antibacterial activity, where the most notable species was *Turbinellus floccosus*, with an MIC = 1000*  μ*g/mL against *Enterococcus faecalis*, while *C*. *violaceovinosus* and *C*. *veraecrucis* showed an MIC = 62.5*  μ*g/mL, MBC = 250*  μ*g/mL, and MIC = 250*  μ*g/mL against *Clavibacter michiganensis*, respectively. Additionally, *Turbinellus floccosus* inhibited the phytopathogenic fungus *Acremonium strictum*, with a PIMG% = 62.20, and also exhibited a PIMG% = 58.73 (*p* ≤ 0.05) against *Colletotrichum asianum*. Regarding toxicity, the three fungal extracts showed moderate toxicity within an LC_50_ range of 100–500 *μ*g/mL against *Artemia salina*. This study provides a first approximation to the potential pharmaceutical and/or agricultural use of extracts of these wild edible mushrooms. The results obtained open the possibility of testing these extracts in plant models (*in vivo*), contributing to the development of future biodegradable pesticides of natural origin.

## 1. Introduction

In recent decades, the excessive and/or inappropriate use of different antimicrobial agents, as well as social and economic factors, has led to populations of different microorganisms becoming increasingly resistant to such substances. Even though this is a natural phenomenon, the process has been accelerated by the excessive use of antibiotics to treat human medical conditions, as well as by their widespread use in the livestock and agricultural industry [1–3]. Therefore, it is not surprising that the World Health Organization (WHO) has recognized multidrug resistance as a major threat to public health and of global concern [2, 3]. Currently, 700,000 people die each year due to antimicrobial resistance, and it is estimated that this number will increase to 10 million by 2050, mainly due to some human pathogenic bacteria [1]. In addition, there is also a need to find new and effective antifungal agents against human pathogenic opportunistic yeasts [4, 5]. Phytopathogenic bacteria and fungi cause devastating damage to a wide range of crops and generate significant pre- and postharvest economic losses, and both types of organisms represent a threat to the sustainability of global food production, causing losses of more than one billion dollars each year worldwide [6–9]. Among the bacteria that cause diseases in practically all crops are *Clavibacter michiganensis*, *Erwinia amylovora*, *Pseudomonas syringae*, *Ralstonia solanacearum*, *Xanthomonas campestris*, *and Xylella* [6, 10, 11]. It is therefore reasonable to note that the appropriate conditions exist for the emergence of “superphytopathogenic” bacteria, just like multidrug-resistant human superbacteria [6], as well as the appearance of more abundant and frequent phytopathogenic fungi, particularly the genera *Fusarium*, *Alternaria*, *Fusicladium*, *Neoerysiphe*, *Mycosphaerella*, *Trichoderma*, and *Epicoccum*, among others [12].

At present, research has focused on exploring strategies for the management and control of human pathogenic and phytopathogenic microorganisms with the purpose of protecting human health and the environment [7, 13]. One of these strategies is the search and discovery of new bioactive compounds present in wild macrofungi, which have been shown to have diverse bioactive properties, as well as high nutritional value and profitability, among other characteristics. Thus, wild macrofungi can be used as alternative organisms for the development of drugs, functional foods, and supplements to combat and prevent human diseases [13–17]. A wide variety of bioactive molecules have been reported in edible wild macrofungi; some examples are polysaccharides (*β*-glucans and lentinan) with immunomodulatory, antitumor, and antibacterial activity; unsaturated fatty acids (oleic and linoleic) with anti-inflammatory and antioxidant properties; proteins (lectins) with antifungal, immunomodulatory, and antiproliferative effects; sterols (ergosterol) as precursors of vitamin D; triterpenes (ganoderic acids) with antiproliferative and antibacterial activity; phenols (flavonoids and phenolic acids) with antioxidant and antibacterial activity; and other metabolites such as strobilurins with antifungal activity [15–18]. Numerous edible and medicinal mushrooms have been studied for their bioactivities, where the most common genera with antimicrobial properties include *Cordyceps*, *Dictyophora*, *Ganoderma*, *Lentinus*, *Pleurotus*, and *Tremella* [17, 18].

There are still gaps to be addressed in the current knowledge of the bioactivities of many edible mushrooms. Species of the genera *Cantharellus* and *Turbinellus*, which belong to the group of wild ectomycorrhizal fungi, are associated with native trees and generate ecological benefits in tropical and temperate forests in eastern Mexico. In addition, their fruiting bodies, which are harvested for consumption and sale during the rainy season, are highly coveted and have high economic value [19–21]. Species such as *Cantharellus cibarius*, *Cantharellus roseocanus*, *Cantharellus veraecrucis*, *Cantharellus violaceovinosus*, and *Turbinellus floccosus* have attracted the attention of researchers from different areas such as taxonomy, ecology, and nutrition [19–24]. However, their potential as a source of nutraceutical and bioactive components with pharmacological applications is still under investigation [22–24]. Therefore, they represent excellent study organisms due to their abundance and distribution in oak and pine forests. Considering the above, this study is aimed at evaluating the antibacterial, antifungal, and acute toxicity activities of crude extracts of *C. veraecrucis*, *C*. *violaceovinosus*, and *Turbinellus floccosus* through *in vitro* assays.

## 2. Materials and Methods

### 2.1. Biological Material

Fresh basidiomes of *C. veraecrucis* and *C. violaceovinosus* were collected during June–October 2021 in the oak forest of the municipality of Zentla (837–850 m.a.s.l.), while basidiomes of *Turbinellus floccosus* were collected in a *Pinus* forest at Cofre de Perote National Park (3000–3500 m.a.s.l.), both located in the central region of the State of Veracruz, Mexico. The basidiomes were collected following an opportunistic sampling protocol [25] and subsequently frozen and lyophilized (LABCONCO, Kansas City, United States).

### 2.2. Fungal Extracts

Dried samples (70 g) of each edible mushroom were macerated separately using a chloroform–methanol mixture (1 : 1 *v*/*v*) to improve the extraction of polar and nonpolar compounds [26, 27]. The samples were kept in an ultrasound bath (Elmasonic S50R, Singen, Germany) for 60 min, with a frequency of 40 kHz. Subsequently, the biomass was separated by vacuum filtration, and the solvent was then evaporated in a rotary evaporator at 40°C (Büchi R-100, Flawil, Switzerland) [28]. Finally, the crude extracts were centrifuged at 3260 × *g* for 10 min, and the resulting supernatant was filtered using 25/0.45 *μ*m Econofilter PTFE filters (Agilent Technologies, Germany). The centrifugation and filtration implemented in this study improved the purity of the extracts, which were stored in vials at 4°C.

### 2.3. Pathogenic Organisms and Culture Conditions

In the case of bacterial strains of medical importance, the Gram-positive bacteria *Enterococcus faecalis* (ATCC 29212) and *Staphylococcus aureus* (ATCC 25923) and Gram-negative bacteria *Escherichia coli* (ATCC 35218) were spread on Müeller–Hinton agar (MCD LAB, Oaxaca, Mexico) and incubated for 24 h at 37°C ± 2°C. In the case of strains of phytopathogenic bacteria, the Gram-positive bacteria *Clavibacter michiganensis* subsp. *michiganensis* (ID/46) and the Gram-negative bacteria *Erwinia persicina* (h-5), *Pseudomonas syringae* (ID/17), *Rhizobium radiobacter* (ID/70), and *Xanthomonas campestris* (ID/138) were provided by the Bacteriology Laboratory of the Servicio Nacional de Sanidad, Inocuidad y Calidad Agroalimentaria (SENASICA) and the Pilot Plant for the Development of Biological Control Agents of the Instituto de Ecología, A.C. The bacteria were incubated on Müeller–Hinton agar plates for 48 h at 25°C ± 2°C. Colonies of the two groups of bacteria were resuspended in Müeller–Hinton broth (Difco, Sparks, Maryland, United States) until obtaining a concentration of 1.5 × 10^5^ CFU/mL [29].

The yeast of clinical interest *Candida tropicalis* (CECT 1440), obtained from the Spanish Type Culture Collection (CECT), was spread on Potato Dextrose Agar (PDA) (MCD LAB, Oaxaca, Mexico) and incubated for 48 h at 35°C ± 2°C. Colonies of the yeast were resuspended in Potato Dextrose Broth (PDB) (Condalab, Madrid, Spain) until reaching a concentration of 1.5 × 10^4^ CFU/mL [5, 30]. The phytopathogenic fungi *Acremonium strictum* (CBF-230), *Colletotrichum asianum* (CBF-338), and *Fusarium oxysporum* f. sp. *lycopersici* (CBF-338) were provided by the Pilot Plant for the Development of Biological Control Agents, Instituto de Ecología, A.C. The phytopathogenic fungi were spread over a PDA medium and incubated for 8 days at 25°C ± 2°C prior to the evaluations to obtain an actively growing mycelium.

### 2.4. Antibacterial Evaluation

Antibacterial activity was evaluated following the guidelines of the Clinical and Laboratory Standards Institute (CLSI) (protocol M7-A9) [29], based on the broth microdilution method, with adaptations to evaluate extracts according to Serrano-Márquez et al. [28], Zengin et al. [31], and Wong and Ramli [32]. The minimum inhibitory concentration (MIC) of the crude extracts against bacterial strains of medical importance and phytopathogenic bacteria was determined. The broth microdilution method makes it possible to obtain a quantitative result (MIC) and determine whether an extract is bacteriostatic or bactericidal [29, 32]. In deep-well plates, serial dilutions of each extract were made using Müeller–Hinton broth until concentrations of 2000–62.5 *μ*g/mL were obtained. Subsequently, in sterile 96-well round-bottom polystyrene microplates (Costar, Kennebunk, Maine, United States), 75 *μ*L of each extract concentration was added and mixed with 75 *μ*L of the bacterial suspension (1.5 × 10^5^ CFU/mL) to obtain a final volume of 150 *μ*L per well and final concentrations from Row A (1000 *μ*g/mL) to Row F (31.25 *μ*g/mL). The wells in Row G were used as negative controls (Müeller–Hinton broth, bacterial suspension, and 10% DMSO) and the wells in Row H as sterility controls (Müeller–Hinton broth). Ampicillin and chloramphenicol (Sigma-Aldrich, St. Louis, Missouri, United States) were used at an initial concentration of 1000–0.4882 *μ*g/mL as positive controls [28]. The microplates were incubated depending on the type of bacteria. After incubation, 30 *μ*L of 3% 2,3,5-triphenyltetrazolium chloride (TTC) (Sigma-Aldrich, St. Louis, Missouri, United States) was added to each well [31]. The lowest concentration of the extract that remained colorless and showed no visible growth was recorded as the MIC. This evaluation was carried out in three events, with each extract evaluated in triplicate in each event. The minimum bactericidal concentration (MBC) was determined in bioactive extracts that had an MIC ≤ 1000*  μ*g/mL. For this, 1 *μ*L was taken from each well and spread on plates with Müeller–Hinton agar, which were subsequently incubated. Following incubation, the lowest concentration that remained without CFU growth corresponded to the MBC [32].

### 2.5. Antiyeast Evaluation

Antiyeast activity was evaluated following protocol M27-A2 of the CLSI guidelines [30], using the broth microdilution method with adaptations to evaluate extracts, as reported by Yu et al. [5], Wong and Ramli [32], and Morales et al. [33]. The MIC and minimum fungicide concentration (MFC) of the crude extracts were determined against *Candida tropicalis*. This assay was similar to the antibacterial assay, but the dilutions of the extract concentrations (2000–62.5 *μ*g/mL) were performed in PDB [5]. In microplates, 75 *μ*L of each extract concentration was mixed with 75 *μ*L of the yeast suspension (1.5 × 10^4^ CFU/mL) to obtain a final volume of 150 *μ*L per well and final concentrations from Row A (1000 *μ*g/mL) to Row F (31.25 *μ*g/mL). A negative control (PDB, fungal suspension, and 1% DMSO) and a sterility control (PDB) were also included. Ketoconazole (Sigma-Aldrich, St. Louis, Missouri, United States) was used at an initial concentration of 1000–0.0610 *μ*g/mL as a positive control. The microplates were incubated and 30 *μ*L of 3% TTC was subsequently added [33]. Replicates were performed, and the MIC was recorded as in the antibacterial activity evaluation. The MFC was determined in bioactive extracts that had an MIC ≤ 1000*  μ*g/mL. For this, 1 *μ*L was taken from each well, streaked onto a PDA medium, and incubated [32].

### 2.6. Antifungal Evaluation

Antifungal activity was evaluated following the method of inhibition of mycelial radial growth (poisoned food technique). This is a common and sensitive technique for evaluating the antifungal properties of extracts and allows the determination of the percentage of inhibition of mycelial growth (PIMG%). The fungal extracts were evaluated according to the method reported by Al-Burtamani et al. [34], Kaur et al. [35], and Ahuja et al. [36]. The PIMG% was determined against phytopathogenic fungi. For this, PDA medium and a stock solution adjusted to a final concentration of 1000 *μ*g/mL (1% DMSO) were poured into a Petri dish (9 cm). Subsequently, a mycelial disc (5 mm in diameter) of each fungus was inoculated in the center of each plate, respectively [34, 35]. PDA medium was used as a negative control and ketoconazole (Sigma-Aldrich, St. Louis, Missouri, United States) as a positive control. The plates were incubated for 8 days at 25°C ± 2°C, and mycelial growth was recorded every 2 days [36]. The evaluation was carried out in triplicate and the results are expressed according Formula (1) [34]:

(1) $$\begin{array}{c} \text{PIMG}\%=\left[\left(\text{IMc}-\text{IMt}\right)/\text{IMc}\right]\times 100 \end{array}$$

where IMc is the growth of the average diameters of the control mycelium and IMt is the growth of the average diameters of the mycelium with treatment.

### 2.7. Determination of Acute Toxicity

Acute toxicity was evaluated following the methods established by Meyer et al. [37] and Nguta and Mbaria [38], with modifications. The genus *Artemia* was used in this bioassay because it allows a preliminary analysis related to toxicity, which is effective in the evaluation of the toxicity of fungi of the Basidiomycota division [39]. Nauplii of *Artemia salina* (White Mountain, Utah, United States) were hatched by placing 50 mg of cysts in 100 mL of artificial marine water (2.8% NaCl) and 2 mg of docosahexaenoic acid (DHA) (Acua Biomar, Mazatlán, Sinaloa, Mexico) as enrichment for the nauplii; this hatching medium was modified from the original method [37]. The cysts were incubated with aeration and constant lighting at 27°C ± 2°C for 24 h. Subsequently, 400 *μ*L of artificial seawater and 10 nauplii were placed per well in 24-well microplates, and stock solutions of each extract were then added, adjusting the concentrations until reaching 1000, 100, and 10 *μ*g/mL [37, 38]. The concentrations were evaluated in triplicate. Artificial marine water (0.5% DMSO) was used as a negative control, and potassium dichromate (K_2_Cr_2_O_7_) was used in the different concentrations (1000, 100, and 10 *μ*g/mL) as a positive control. The microplates were incubated at 27°C ± 2°C for 24 h. After incubation, the mean lethal concentration (LC_50_) was determined, as well as the mortality percentage of the nauplii according to Formula (2) [38]:

(2) $$\begin{array}{c} \text{Mortality}\%=\left[\left(\text{total deaths}\right)/\left(\text{total }Artemia\right)\right]\times 100. \end{array}$$

### 2.8. Statistical Analysis

The antifungal activity data are shown as the mean ± SD (*n* = 3) and were analyzed with a one-way analysis of variance (ANOVA) to determine statistically significant differences between the means of the three edible mushrooms. Specific differences in PIMG% between fungal species were determined with a Tukey's post hoc test *p* ≤ 0.05 [36]. This analysis was performed in JMP Version 14. To determine the LC_50_, the Probit method was used with a 95% confidence interval. The Probit method consists of a linear regression to establish the relationship between the concentration of a toxic substance and the response of the tested species exposed to the toxicant for a given time [37]. These data were processed in SPSS Version 25.

## 3. Results and Discussion

### 3.1. Antibacterial and Antiyeast Activity

In the first evaluation, the MIC, MBC, and MFC of the crude extracts were determined against three bacterial species and one yeast species of clinical interest. In this assay, one fungal extract showed bioactivity, and the level of bioactivity was defined according to O'Donnell et al. [40], ranging from no bioactivity (MIC > 1000*  μ*g/mL) to slight bioactivity (MIC 501–1000 *μ*g/mL), moderate bioactivity (MIC 126–500 *μ*g/mL), good bioactivity (MIC 26–125 *μ*g/mL), strong bioactivity (MIC 10–25 *μ*g/mL), and very strong bioactivity (MIC < 10*  μ*g/mL). In the case of the microorganisms of clinical interest (see Table 1), the extract of *Turbinellus floccosus* inhibited the growth of the Gram-positive bacterium *Enterococcus faecalis* (ATCC 29212), with MIC values = 1000*  μ*g/mL, exhibiting slight bioactivity [26, 40]. To our knowledge, this is the first report of antibacterial activity in *Turbinellus floccosus* against a bacterium of clinical interest. It should be noted that the bacterium *Enterococcus faecalis* is part of the human microbiota but can cause serious infections in the urinary tract, endocarditis, bacteremia, and wound infections [22]. Only antioxidant activity had been previously reported in *Turbinellus floccosus* extracts [23].

*C. veraecrucis* and *C*. *violaceovinosus* did not inhibit the growth of bacteria of clinical interest. However, species of the genus *Cantharellus* are considered highly sought-after foods [20], and perhaps the most prominent species of the genus is *Cantharellus cibarius* [22, 39]. There is notable evidence of the antibacterial activity of this species. For example, Kozarski et al. [22] reported the activity of the methanol extract of *Cantharellus cibarius* against *Enterococcus faecalis*, with an MIC = 156*  μ*g/mL and MBC = 2500*  μ*g/mL. Similarly, Kolundžić et al. [41] reported that the methanol extract of *Cantharellus cibarius* was active against *Enterococcus faecalis*, with an MIC = 125*  μ*g/mL. Tamrakar et al. [42] found that *Cantharellus ferruginascens* has inhibitory effects against *Staphylococcus aureus*, with an MIC = 100*  μ*g/mL. Muszyńska et al. [43] reported that the methanol extract of *Cantharellus cibarius* had a slight antimicrobial activity against *Staphylococcus aureus*. However, methanol extracts of *Cantharellus tubaeformis* and *Cantharellus cibarius* did not show antimicrobial activity against *Staphylococcus aureus*, *Escherichia coli*, and *Candida albicans* [44, 45]. In another study, Santoyo et al. [46] reported that the methanol extract of *Cantharellus cibarius* did not show fungicidal activity against *Candida albicans*, similar to our results, although it is a different yeast than the one evaluated in our study, but of clinical interest. The available evidence [22, 41, 47] suggests that wild mushroom extracts are more active against Gram-positive bacteria than Gram-negative bacteria. Although our results showed slight bioactivity, they are not consistent enough to support this inference. Differences in the cell wall structure of Gram-positive and Gram-negative bacteria could partly explain the different inhibitory effects of wild mushroom extracts [47]. On the other hand, yeast cells (eukaryote) and bacterial cells (prokaryote) differ in several aspects, which may explain the lack of bioactivity against *Candida tropicalis* in our extracts. Yeasts are larger in size and exhibit a different type of reproduction (budding) and different structures (cell membranes with ergosterol and walls with chitin), which may confer resistance to these extracts. Antifungal agents generally have a site of action in the fungal membrane and wall, particularly in the biosynthesis of ergosterol, glucans, microtubules, and mannoproteins [4, 48, 49]. It should be noted that solvents for extraction can modify the bioactivity, quality, quantity, and safety of the expected bioproducts; the solvents used in the present study (chloroform–methanol mixture) can dissolve polar and nonpolar compounds [18, 26, 27, 50]. The effectiveness of different solvents is related to the chemical nature and solubility of the bioactive compounds present in macrofungi. Therefore, the choice of solvent is crucial for extracting higher or lower quantities of said compounds, which can potentially modify the expected results. Antimicrobial activity has been reported in extracts of various polarities obtained mainly with ethanol, methanol, water, ethyl acetate, and chloroform [50].

In the case of phytopathogenic bacteria, the extracts of the three edible mushrooms showed inhibition against at least one species (see Table 2). The most notable results were observed with the extract of *C. violaceovinosus*, which inhibited the growth of *Clavibacter michiganensis* (ID/46) with good bioactivity (MIC = 62.5*  μ*g/mL and MBC = 250*  μ*g/mL) and was also moderately bioactive (MIC = 500*  μ*g/mL) against *Pseudomonas syringae* (ID/17). These results are consistent with those reported by Cieniecka-Rosłonkiewicz et al. [51], where *Cantharellus cibarius* extracts were more active against phytopathogenic bacteria than against human pathogenic bacteria.

The results of the inhibition of the Gram-positive bacteria *Clavibacter michiganensis* are also consistent with those reported by Espinosa-García et al. [52], where chloroform–methanol extracts of different strains of the genus *Ganoderma* were active (MIC = 31.5–1000 *μ*g/mL) against this phytopathogenic bacterial species responsible for tomato canker. However, we obtained different results in the case of the extracts of the two *Cantharellus* species, as these were active against *Pseudomonas syringae* (ID/17) and the extracts of the *Ganoderma* strains were not [52]. However, chloroform and ethanol extracts of *Cantharellus cibarius* have shown inhibition against the Gram-negative bacteria *Xanthomonas campestris*, but they were evaluated by the disk diffusion method [53].

In the antibacterial activity tests against phytopathogenic bacteria and bacteria of clinical interest (Gram-positive and Gram-negative), we used as positive controls the antibiotic ampicillin, whose mechanism of action inhibits cell wall synthesis, and chloramphenicol, which inhibits protein synthesis. In comparison, we can assume that our bioactive extracts have a similar mechanism of action and a short-medium activity spectrum, since they inhibited the growth of the two types of bacteria evaluated [1, 3]. We can also confirm that the differences in antimicrobial activity observed between our study and previous results reported by several authors for species of the same genus are largely due to the origin of the wild fungi, the extraction solvent, the evaluation method, the concentrations, and the bacterial strain [50, 54].

### 3.2. Antifungal Activity

**Figure 1:**
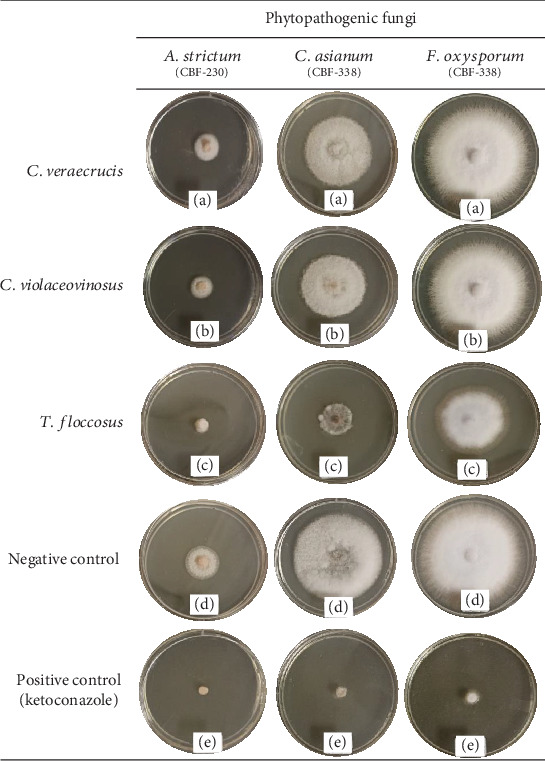
*In vitro* evaluation of crude extracts of *Cantharellus veraecrucis* (a), *C*. *violaceovinosus* (b), *Turbinellus floccosus* (c), negative control (d), and positive control (e) against phytopathogenic fungi at 8 days.

The antifungal potential of the crude extracts of the three species of edible mushrooms studied was evaluated at a concentration of 1000 *μ*g/mL [35] against three phytopathogenic fungi. The growth of the mycelial diameter was recorded every 2 days and the PIMG(%) was determined. The results are shown in Table 3 and Figure 1. The most notable results were obtained with the extract of *Turbinellus floccosus*, which inhibited two of the three strains of phytopathogenic fungi, with percentages greater than 50%. It exhibited a PIMG% = 62.20 (*p* ≤ 0.05) against the species *Acremonium strictum* (CBF-230), where a value of *p* ≤ 0.05 indicates statistically significant differences compared to the extracts of the other two edible mushrooms evaluated. It also showed a PIMG% = 58.73 (*p* ≤ 0.05) against *Colletotrichum asianum* (CBF-338) and a PIMG% = 30.09 against *Fusarium oxysporum*.

The results obtained in this evaluation provide new insights about the effects of edible mushroom extracts against phytopathogenic fungi. To date, there is no information available on the antifungal activity of these three fungal species belonging to the Cantharellaceae family. Little research has been done on the antifungal activity of wild mushrooms against phytopathogenic fungi. While Imtiaj et al. [55] reported an antifungal activity of 41.73% against *Colletotrichum gloeosporioides* using extracts of *Stereum ostrea*, the extracts from *Turbinellus floccosus* used in our study exhibited even higher inhibition percentages against two strains of phytopathogenic fungi. Similarly, Owaid et al. [56] investigated the antifungal activity of *Pleurotus* spp. against *Trichoderma harzianum* and *Verticillium* sp. and obtained a PIMG% = 11.60 and 12.33, respectively. These percentages of inhibition are lower than those obtained in our study. Furthermore, Imtiaj and Lee [57] reported the activity of some wild mushrooms against *Botrytis cinerea*, *Colletotrichum gloeosporioides*, and *Colletotrichum miyabeanus*, with a PIMG% between 12.07 and 81.33, which is consistent with our results, confirming that wild mushrooms are active against phytopathogenic fungi. Filamentous fungi and yeasts share a similar cellular structure but differ in shape, reproduction, growth rate, and respiration, which may explain why the extracts were active against filamentous fungi but not against yeasts. We used the antifungal ketoconazole as a positive control, which is a broad-spectrum azole that inhibited both yeasts and filamentous fungi, and thus, our extracts would be considered to have a narrow spectrum, as they only inhibited the growth of phytopathogenic fungi [4, 48, 49].

Within the context of pest control, natural forest areas are a potential source of alternative agents of biological origin and low environmental impact. Thus, wild mushrooms are a promising option given their high diversity and adaptability to different conditions. However, they still remain underexplored. A strategy to facilitate the identification of wild fungi with antimicrobial activity could consider their ecological role and whether they are used locally. The three species of edible ectomycorrhizal fungi evaluated in the present study are an attractive prospect for this purpose. Two of them (*C. violaceovinosus* and *C*. *veraecrucis*) are new to science [20, 21] and adapted to the conditions of tropical *Quercus* forests in Mexico. To date, there is only one study [24] that has provided an approximation of the chemical composition of *C. violaceovinosus* and *C*. *veraecrucis*, identifying valuable nutraceutical compounds such as phenols, ascorbic acid, *β*-carotenoids, and carbohydrates, which may provide bioactive properties that could inhibit the growth of bacterial and fungal phytopathogens. Moreover, other species, such as *Cantharellus cibarius*, have shown promising antihypertensive, cytotoxic, antioxidant, and antibacterial bioactivity [22]. In addition, González-Morales et al. [23] reported the antioxidant activity of extracts of the species *Turbinellus floccosus* and also made a preliminary identification of some groups of compounds such as flavonoids, saponins, tannins, quinones, and coumarins, which could be related to the antibacterial and antifungal activity that *Turbinellus floccosus* showed in our study. Basidiomycete fungi produce extracts and compounds with antibacterial and antifungal activity [50], where some examples of compounds with dual activity are dentifragilin A, striatal D [58], dentipellin, erinacine A–C [59], griseococcin [60], and microporenic acid A, D, and E [61].

The prospects that arise after analyzing the outstanding results of the antibacterial and antifungal activity of the extracts of *C. veraecrucis*, *C*. *violaceovinosus*, and *Turbinellus floccosus* include testing the extracts on sick plants in the field in order to contribute to the development of future biodegradable pesticides of natural origin. The next step will be to isolate or identify the metabolites responsible for said activity through metabolomic profiles and chromatographic techniques (HPLC, GC-MS, and NMR), all this with the aim of contributing to the continuous bioprospecting of new sources of organisms for their sustainable use for the benefit of the scientific community, the general population, and agricultural producers, which will add value to the edible mushrooms studied, increasing interest in them and encouraging the conservation of their natural habitats.

### 3.3. Acute Toxicity

Acute toxicity was assessed by both the mortality percentage and LC_50_ of each of the three species of edible mushrooms using the model organism *Artemia salina* (see Table 4). The toxicity level of the extracts in this assay was defined according to Nguta et al. [62], where LC_50_ > 1000 *μ*g/mL indicates no toxicity, LC_50_ 500–1000 *μ*g/mL indicates weak toxicity, LC_50_ 100–500 *μ*g/mL indicates moderate toxicity, and LC_50_ < 100 *μ*g/mL indicates high toxicity. All three fungal extracts showed toxicity within an LC_50_ range of 100–500 *μ*g/mL, indicating moderate toxicity. It should be noted that the extract that exhibited the lowest toxicity was that of *C. violaceovinosus*, with an LC_50_ of 166.238 *μ*g/mL. According to Oyetayo et al. [63], the tolerance of *Artemia salina* to fungal extracts at concentrations above 100 *μ*g/mL indicates that these extracts are safe to use.

In a previous study by Ruiz-González et al. [39], an aqueous extract of *Cantharellus cibarius* showed an LC_50_ > 1000 *μ*g/mL when tested on nauplii of *Artemia franciscana*, which is a less toxic concentration than that reported in our results. This difference is likely due to the type of extraction and the species of *Artemia* evaluated. However, Kidukuli et al. [64] evaluated methanol extracts of the species *Cantharellus platyphyllus* and *Cantharellus isabellinus* and obtained LC_50_ values of 7.85 and 17.35 *μ*g/mL, respectively, which are more toxic concentrations than those shown by the two species of *Cantharellus* evaluated in our study. In another study by Ugbogu et al. [65], species of the genus *Cantharellus* were found to be highly nutritious and nontoxic to rats at the doses tested and can be therefore used for medicinal and culinary purposes.

It is worth mentioning that, in the continuous search for drugs, functional foods, and natural products, their potential toxicity must be considered when ingested at high doses or in combination with other drugs, and it is thus advisable to subject them to safety toxicological evaluations [66]. Certain mushroom species can be toxic; some of which are lethal if consumed. Therefore, preliminary toxicity studies are necessary to validate their safety. It is also advisable to determine the species that can be harmful and the recommended state in which they should be consumed (raw or cooked) [15, 17]. In addition, it is recommended to perform the test with *Artemia* from the crude extract stage, which involves the use of mixtures of various chemical compounds; some of which may be potentially toxic [37]. Alves et al. [54] also mention that edible mushroom species used in studies should not cause toxicity when ingested. However, the toxicity of their extracts and individual compounds must be evaluated to gain further insights.

## 4. Conclusions

In conclusion, this study demonstrated that extracts from *C. veraecrucis*, *C*. *violaceovinosus*, and *Turbinellus floccosus* possess promising antibacterial and antifungal properties while exhibiting moderate toxicity levels, suggesting their potential for pharmaceutical and agricultural applications. The results obtained provide a specific advance in the study of edible species of wild mushrooms present in the tropics and subtropics of eastern Mexico. Prior to our study, there was a total lack of research on the bioactivity of these mushroom species, but our results showed that they are bioactive against human pathogens and phytopathogens. This study opens the way to future research where fungal extracts are evaluated in other *in vitro* bioassays and raises the possibility of continuing with the identification of bioactive metabolites responsible for bioactivity. Based on our findings, the next step is to move on to the experimental phase by testing extracts or metabolites against phytopathogenic bacteria and fungi *in vivo* in plants (field phase).

## Figures and Tables

**Table 1:** Antimicrobial activity of extracts of three edible mushrooms against microorganisms of clinical interest.

**Species**	**MIC, MBC, and MFC (*μ*g/mL) against human pathogenic bacteria and yeasts**							
	***E*. *faecalis*** ^**(ATCC 29212)**^		***S. aureus*** ^**(ATCC 25923)**^		***E. coli*** ^**(ATCC 35218)**^		***C. tropicalis*** ^**(CECT 1440)**^	
	**MIC**	**MBC**	**MIC**	**MBC**	**MIC**	**MBC**	**MIC**	**MFC**
*C*. *veraecrucis*	> 1000	> 1000	> 1000	> 1000	> 1000	> 1000	> 1000	> 1000
*C*. *violaceovinosus*	> 1000	> 1000	> 1000	> 1000	> 1000	> 1000	> 1000	> 1000
*T*. *floccosus*	**1000**	> 1000	> 1000	> 1000	> 1000	> 1000	> 1000	> 1000
Ampicillin	1.9531	7.8125	15.625	31.25	15.625	31.25	NA	NA
Chloramphenicol	7.8125	62.5	15.625	125	7.8125	62.5	NA	NA
Ketoconazole	NA	NA	NA	NA	NA	NA	0.1220	0.2441

*Note:* Values of the extracts that show bioactivity are indicated in bold.

Abbreviations: MBC, minimum bactericidal concentration; MFC, minimum fungicide concentration; MIC, minimum inhibitory concentration; NA, not applicable.

**Table 2:** Antibacterial activity of extracts of three edible mushrooms against bacteria of agricultural interest.

**Species**	**MIC and MBC (*μ*g/mL) against phytopathogenic bacteria**									
	***C*. *michiganensis*** ^**(ID/46)**^		***P*. *syringae*** ^**(ID/17)**^		***R. radiobacter*** ^**(ID/70)**^		***X. campestris*** ^**(ID/138)**^		***E. persicina*** ^**(h-5)**^	
	**MIC**	**MBC**	**MIC**	**MBC**	**MIC**	**MBC**	**MIC**	**MBC**	**MIC**	**MBC**
*C*. *veraecrucis*	**250**	> 1000	**1000**	> 1000	> 1000	> 1000	> 1000	> 1000	> 1000	> 1000
*C*. *violaceovinosus*	**62.5**	**250**	**500**	> 1000	> 1000	> 1000	> 1000	> 1000	> 1000	> 1000
*T*. *floccosus*	**1000**	> 1000	> 1000	> 1000	> 1000	> 1000	> 1000	> 1000	> 1000	> 1000
Ampicillin	< 0.4882	< 0.4882	15.625	31.25	250	500	> 1000	> 1000	< 0.4882	< 0.4882
Chloramphenicol	3.9062	3.9062	31.25	31.25	15.625	15.625	250	250	1.9531	1.9531

*Note:* Values of the extracts that show bioactivity are indicated in bold.

Abbreviations: MBC, minimum bactericidal concentration; MIC, minimum inhibitory concentration; NA, not applicable.

**Table 3:** Antifungal activity of extracts of three edible mushrooms against fungi of agricultural importance.

**Species**	**Days**	**Mycelial diameter (mm)**			**PIMG%**		
		***A. strictum*** ^**(CBF-230)**^	***C. asianum*** ^**(CBF-338)**^	***F. oxysporum*** ^**(CBF-338)**^	***A*. *strictum*** ^**(CBF-230)**^	***C*. *asianum*** ^**(CBF-338)**^	***F*. *oxysporum*** ^**(CBF-338)**^
*C*. *veraecrucis*	2	5.00 ± 0.00	6.83 ± 0.29	7.00 ± 0.50	6.25	6.82	8.70^a^
	4	11.00 ± 0.00	24.33 ± 0.29	30.33 ± 0.58	10.81^b^	22.34	21.55^b^
	6	17.17 ± 0.29	38.33 ± 0.76	47.67 ± 0.58	12.71	22.82^c^	21.43^c^
	8	24.67 ± 0.29	52.33 ± 1.15	62.50 ± 0.50	9.76^d^	23.04^d^	13.19^d^

*C*. *violaceovinosus*	2	5.00 ± 0.00	6.83 ± 0.29	7.17 ± 0.29	6.25^a^	6.82	6.52
	4	10.67 ± 0.29	23.33 ± 0.29	33.67 ± 0.29	13.51^b^	25.53	12.93
	6	17.33 ± 0.29	36.17 ± 0.29	49.67 ± 0.58	11.86^c^	27.18^c^	18.13^c^
	8	21.17 ± 0.29	50.67 ± 0.58	64.33 ± 0.58	22.56^d^	25.49^d^	10.65^d^

*T*. *floccosus*	2	5.00 ± 0.00	6.67 ± 0.29	7.00 ± 0.00	6.25^a^	9.09^a^	8.70^a^
	4	7.00 ± 0.87	13.83 ± 0.58	22.67 ± 0.58	43.24^b^	55.85	41.38^b^
	6	8.67 ± 1.26	21.83 ± 0.29	37.23 ± 1.08	55.93	56.04^c^	38.63^c^
	8	10.33 ± 0.58	28.07 ± 0.40	50.33 ± 0.76	62.20	58.73	30.09^d^

Negative control	2	5.33 ± 0.29	7.33 ± 0.29	7.67 ± 0.58	ND	ND	ND
	4	12.33 ± 0.29	31.33 ± 0.29	38.67 ± 0.58	ND	ND	ND
	6	19.67 ± 0.58	49.67 ± 1.15	60.67 ± 1.53	ND	ND	ND
	8	27.33 ± 0.29	68.00 ± 1.73	72.00 ± 1.00	ND	ND	ND

Positive control (ketoconazole)	2	5.0 ± 0.00	5.0 ± 0.00	5.0 ± 0.00	6.25	31.82	34.78
	4	5.0 ± 0.00	5.0 ± 0.00	5.0 ± 0.00	59.46	84.04	87.07
	6	5.0 ± 0.00	5.0 ± 0.00	5.0 ± 0.00	74.58	89.93	91.76
	8	5.0 ± 0.00	5.0 ± 0.00	5.0 ± 0.00	81.71	92.65	93.06

*Note:* Mycelial diameter (mean ± SD); PIMG%, percentage of inhibition of mycelial growth; PIMG% values followed by different letters are significantly different at *p* ≤ 0.05 (ANOVA, Tukey test).

Abbreviation: ND, not detected.

**Table 4:** Acute toxicity of extracts of three edible mushrooms tested on *A*. *salina*.

**Species**	**Total *A*. *salina***	**% mortality**			**LC **_**50**_ ** (*μ*g/mL)**
		**10 *μ*g/mL**	**100 *μ*g/mL**	**1000 *μ*g/mL**	
*C. veraecrucis*	30	6.7	23.3	100	142.197
*C. violaceovinosus*	30	3.3	20	100	166.238
*T*. *floccosus*	30	6.7	26.7	100	132.705
Potassium dichromate	30	36.7	96.7	100	14.343
Artificial marine water + DMSO	30	0	0	0	ND

*Note:* LC_50_ (microgram/milliliter) means lethal concentration.

Abbreviation: ND, not detected.

## Data Availability

The data used to support the findings of this study are included in this article.

## References

[1] Mancuso G., Midiri A., Gerace E., Biondo C. (2021). Bacterial Antibiotic Resistance: The Most Critical Pathogens. *Pathogens*.

[2] World Health Organization (WHO) (2023). Antimicrobial Resistance. https://www.who.int/news-room/fact-sheets/detail/antimicrobial-resistance.

[3] Baran A., Kwiatkowska A., Potocki L. (2023). Antibiotics and Bacterial Resistance—A Short Story of an Endless Arms Race. *International Journal of Molecular Sciences*.

[4] Lee Y., Puumala E., Robbins N., Cowen L. E. (2021). Antifungal Drug Resistance: Molecular Mechanisms in *Candida albicans* and Beyond. *Chemical Reviews*.

[5] Yu H., Lin Z. X., Xiang W. L. (2022). Antifungal Activity and Mechanism of D-Limonene Against Foodborne Opportunistic Pathogen Candida Tropicalis. *LWT*.

[6] Martins P. M. M., Merfa M. V., Takita M. A., De Souza A. A. (2018). Persistence in Phytopathogenic Bacteria: Do We Know Enough?. *Frontiers in Microbiology*.

[7] Khan R. A. A., Najeeb S., Hussain S., Xie B., Li Y. (2020). Bioactive Secondary Metabolites From *Trichoderma* spp. Against Phytopathogenic Fungi. *Microorganisms*.

[8] Ali Q., Zheng H., Rao M. J. (2022). Advances, Limitations, and Prospects of Biosensing Technology for Detecting Phytopathogenic Bacteria. *Chemosphere*.

[9] López-Anchondo A. N., López-de la Cruz D., Gutiérrez-Reyes E., Castañeda-Ramírez J. C., De la Fuente-Salcido N. M. (2021). Antifungal Activity *In Vitro* and *In Vivo* of Mesquite Extract (*Prosopis glandulosa*) Against Phytopathogenic Fungi. *Indian Journal of Microbiology*.

[10] Sundin G. W., Wang N. (2018). Antibiotic Resistance in Plant-Pathogenic Bacteria. *Annual Review of Phytopathology*.

[11] Islam T., Haque M. A., Barai H. R., Istiaq A., Kim J. J. (2024). Antibiotic Resistance in Plant Pathogenic Bacteria: Recent Data and Environmental Impact of Unchecked Use and the Potential of Biocontrol Agents as an Eco-Friendly Alternative. *Plants*.

[12] Li P., Tedersoo L., Crowther T. W. (2023). Global Diversity and Biogeography of Potential Phytopathogenic Fungi in a Changing World. *Nature Communications*.

[13] Li A. P., He Y. H., Zhang S. Y., Shi Y. P. (2022). Antibacterial Activity and Action Mechanism of Flavonoids Against Phytopathogenic Bacteria. *Pesticide Biochemistry and Physiology*.

[14] Radhajeyalakshmi R., Velazhahan R., Prakasam V. (2012). *In Vitro* Evaluation of Solvent Extracted Compounds From Edible Macromycetes Against Phytopathogenic Fungi. *Archives of Phytopathology and Plant Protection*.

[15] Rathore H., Prasad S., Sharma S. (2017). Mushroom Nutraceuticals for Improved Nutrition and Better Human Health: A Review. *PharmaNutrition*.

[16] Sivanandhan S., Khusro A., Paulraj M. G., Ignacimuthu S., Al-Dhabi N. A. (2017). Biocontrol Properties of Basidiomycetes: An Overview. *Journal of Fungi*.

[17] Niego A. G., Rapior S., Thongklang N. (2021). Macrofungi as a Nutraceutical Source: Promising Bioactive Compounds and Market Value. *Journal of Fungi*.

[18] Shen H. S., Shao S., Chen J. C., Zhou T. (2017). Antimicrobials From Mushrooms for Assuring Food Safety. *Comprehensive Reviews in Food Science and Food Safety*.

[19] Lamus V., Franco S., Montoya L., Endara A. R., Caballero L. A., Bandala V. M. (2015). Mycorrhizal Synthesis of the Edible Mushroom *Turbinellus floccosus* With *Abies religiosa* From Central Mexico. *Mycoscience*.

[20] Herrera M., Bandala V. M., Montoya L. (2018). *Cantharellus violaceovinosus*, a New Species From Tropical *Quercus* Forests in Eastern Mexico. *MycoKeys*.

[21] Montoya L., Herrera M., Bandala V. M., Ramos A. (2021). Two New Species and a New Record of Yellow *Cantharellus* From Tropical *Quercus* Forests in Eastern Mexico With the Proposal of a New Name for the Replacement of *Craterellus confluens*. *MycoKeys*.

[22] Kozarski M., Klaus A., Vunduk J. (2015). Nutraceutical Properties of the Methanolic Extract of Edible Mushroom *Cantharellus cibarius* (Fries): Primary Mechanisms. *Food & Function*.

[23] González-Morales A., Ribas-Aparicio R. M., Burrola-Aguilar C. (2021). Actividad Antioxidante de Hongos Silvestres Consumidos Tradicionalmente en el Centro de México. *Scientia Fungorum*.

[24] Hernández-Marañón E. J., Torres A. A., Chen J. (2023). Nutritional and Nutraceutical Components of Four *Cantharellus* Species (Cantharellaceae, Cantharellales) From the Mountain Region, Veracruz, Mexico. *Acta Botánica Mexicana*.

[25] O’Dell T. E., Lodge D. J., Mueller G. M., Mueller G. M., Bills G. F., Foster M. S. (2004). Approaches to Sampling Macrofungi. *Biodiversity of Fungi, Inventory and Monitoring Methods*.

[26] Doğan H. H., Duman R., Özkalp B., Aydin S. (2013). Antimicrobial Activities of Some Mushrooms in Turkey. *Pharmaceutical Biology*.

[27] Salem M. A., Jüppner J., Bajdzienko K., Giavalisco P. (2016). Protocol: A Fast, Comprehensive and Reproducible One-Step Extraction Method for the Rapid Preparation of Polar and Semi-Polar Metabolites, Lipids, Proteins, Starch and Cell Wall Polymers From a Single Sample. *Plant Methods*.

[28] Serrano-Márquez L., Trigos Á., Couttolenc A., Padrón J. M., Shnyreva A. V., Mendoza G. (2021). Antiproliferative and Antibacterial Activity of Extracts of *Ganoderma* Strains Grown *In Vitro*. *Food Science and Biotechnology*.

[29] CLSI (2012). *Methods for Dilution Antimicrobial Susceptibility Tests for Bacteria that Grow Aerobically; Approved Standard - Ninth Edition. CLSI Document M07-A9*.

[30] CLSI (2002). *Reference Method for Broth Dilution Antifungal Susceptibility Testing of Yeasts; Approved Standard - Second Edition. CLSI Document M27-A2*.

[31] Zengin G., Sarikurkcu C., Gunes E. (2015). Two *Ganoderma* Species: Profiling of Phenolic Compounds by HPLC-DAD, Antioxidant, Antimicrobial and Inhibitory Activities on Key Enzymes Linked to Diabetes Mellitus, Alzheimer’s Disease and Skin Disorders. *Food & Function*.

[32] Wong J. X., Ramli S. (2021). Antimicrobial Activity of Different Types of *Centella asiatica* Extracts Against Foodborne Pathogens and Food Spoilage Microorganisms. *LWT*.

[33] Morales G., Paredes A., Sierra P., Loyola L. A. (2008). Antimicrobial Activity of Three *Baccharis* Species Used in the Traditional Medicine of Northern Chile. *Molecules*.

[34] Al-Burtamani S. K. S., Fatope M. O., Marwah R. G., Onifade A. K., Al-Saidi S. H. (2005). Chemical Composition, Antibacterial and Antifungal Activities of the Essential Oil of *Haplophyllum tuberculatum* From Oman. *Journal of Ethnopharmacology*.

[35] Kaur M., Goel M., Mishra R. C., Lahane V., Yadav A. K., Barrow C. J. (2023). Characterization of the Red Biochromes Produced by the Endophytic Fungus *Monascus purpureus* CPEF02 With Antimicrobial and Antioxidant Activities. *Fermentation*.

[36] Ahuja R., Sidhu A., Bala A. (2019). Synthesis and Evaluation of Iron(ii) Sulfide Aqua Nanoparticles (FeS-NPs) Against *Fusarium verticillioides* Causing Sheath Rot and Seed Discoloration of Rice. *European Journal of Plant Pathology*.

[37] Meyer J. L., Ferrigni B. N., Putnam N. R., Jacobsen L. B., Nichols D. E., McLaughlin J. L. (1982). Brine Shrimp: A Convenient General Bioassay for Active Plant Constituents. *Planta Medica*.

[38] Nguta J. M., Mbaria J. M. (2013). Brine Shrimp Toxicity and Antimalarial Activity of Some Plants Traditionally Used in Treatment of Malaria in Msambweni District of Kenya. *Journal of Ethnopharmacology*.

[39] Ruiz-González L. E., Guzmán-Dávalos L., Guerrero-Galván S. R., Vega-Villasante F. (2023). Mushrooms to Live or Die: Toxicity of Some Basidiomycota Using *Artemia franciscana*. *Brazilian Journal of Biology*.

[40] O’Donnell F., Smyth T. J. P., Ramachandran V. N., Smyth W. F. (2010). A Study of the Antimicrobial Activity of Selected Synthetic and Naturally Occurring Quinolines. *International Journal of Antimicrobial Agents*.

[41] Kolundžić M., Stanojković T., Radović J. (2017). Cytotoxic and Antimicrobial Activities of *Cantharellus cibarius* Fr. (Cantarellaceae). *Journal of Medicinal Food*.

[42] Tamrakar S., Nishida M., Amen Y. (2017). Antibacterial Activity of Nepalese Wild Mushrooms Against *Staphylococcus aureus* and *Propionibacterium acnes*. *Journal of Wood Science*.

[43] Muszyńska B., Kała K., Firlej A., Sułkowska-Ziaja K. (2016). *Cantharellus cibarius*: Culinary-Medicinal Mushroom Content and Biological Activity. *Acta Poloniae Pharmaceutica*.

[44] Keller C., Maillard M., Keller J., Hostettmann K. (2002). Screening of European Fungi for Antibacterial, Antifungal, Larvicidal, Molluscicidal, Antioxidant and Free-Radical Scavenging Activities and Subsequent Isolation of Bioactive Compounds. *Pharmaceutical Biology*.

[45] Dimitrijevic M. V., Mitic V. D., Nikolic J. S. (2019). First Report About Mineral Content, Fatty Acids Composition and Biological Activities of Four Wild Edible Mushrooms. *Chemistry & Biodiversity*.

[46] Santoyo S., Ramírez-Anguiano A. C., Reglero G., Soler-Rivas C. (2009). Improvement of the Antimicrobial Activity of Edible Mushroom Extracts by Inhibition of Oxidative Enzymes. *International Journal of Food Science & Technology*.

[47] Zavastin D. E., Bujor A., Tuchiluş C. (2016). Studies on Antioxidant, Antihyperglycemic and Antimicrobial Effects of Edible Mushrooms *Boletus edulis* and *Cantharellus cibarius*. *Journal of Plant Development*.

[48] Odds F. C., Brown A. J., Gow N. A. R. (2003). Antifungal Agents: Mechanisms of Action. *Trends in Microbiology*.

[49] Mazu T. K., Bricker B. A., Flores-Rozas H., Ablordeppey S. Y. (2016). The Mechanistic Targets of Antifungal Agents: An Overview. *Mini Reviews in Medicinal Chemistry*.

[50] Lysakova V., Krasnopolskaya L., Yarina M., Ziangirova M. (2024). Antibacterial and Antifungal Activity of Metabolites From Basidiomycetes: A Review. *Antibiotics*.

[51] Cieniecka-Rosłonkiewicz A., Sas A., Przybysz E., Morytz B., Syguda A., Pernak J. (2009). Ionic Liquids for the Production of Insecticidal and Microbicidal Extracts of the Fungus *Cantharellus cibarius*. *Chemistry & Biodiversity*.

[52] Espinosa-García V., Mendoza G., Shnyreva A. V., Padrón J. M., Trigos Á. (2021). Biological Activities of Different Strains of the Genus *Ganoderma* spp. (Agaricomycetes) From Mexico. *International Journal of Medicinal Mushrooms*.

[53] Dulger B., Gonuz A., Gucin F. (2004). Antimicrobial Activity of the Macrofungus *Cantharellus cibarius*. *Pakistan Journal of Biological Sciences*.

[54] Alves M. J., Ferreira I. C. F. R., Martins A., Pintado M. (2012). Antimicrobial Activity of Wild Mushroom Extracts Against Clinical Isolates Resistant to Different Antibiotics. *Journal of Applied Microbiology*.

[55] Imtiaj A., Jayasinghe C., Lee G. W., Lee T. S. (2007). Antibacterial and Antifungal Activities of *Stereum ostrea*, an Inedible Wild Mushroom. *Mycobiology*.

[56] Owaid N., Al Saeedi S. S. S., Abed I. A., Shahbazi P., Sabaratnam V. (2016). Antifungal Activities of Some *Pleurotus* Species (Higher Basidiomycetes). *Walailak Journal of Science and Technology (WJST)*.

[57] Imtiaj A., Lee T. S. (2007). Screening of Antibacterial and Antifungal Activities From Korean Wild Mushrooms. *World Journal of Agricultural Sciences*.

[58] Sum W. C., Mitschke N., Schrey H. (2022). Antimicrobial and Cytotoxic Cyathane-Xylosides From Cultures of the Basidiomycete *Dentipellis fragilis*. *Antibiotics*.

[59] Ha L. S., Ki D. W., Kim J. Y., Choi D. C., Lee I. K., Yun B. S. (2021). Dentipellin, a New Antibiotic From Culture Broth of *Dentipellis fragilis*. *Journal of Antibiotics*.

[60] Ye Y., Zeng Q., Zeng Q. (2020). Griseococcin (1) From *Bovistella radicata* (Mont.) Pat and Antifungal Activity. *BMC Microbiology*.

[61] Chepkirui C., Yuyama K. T., Wanga L. A. (2018). Microporenic Acids A–G, Biofilm Inhibitors, and Antimicrobial Agents From the Basidiomycete *Microporus* Species. *Journal of Natural Products*.

[62] Nguta J. M., Mbaria J. M., Gakuya D. W., Gathumbi P. K., Kabasa J. D., Kiama S. G. (2011). Biological Screening of Kenyan Medicinal Plants Using *Artemia salina* L. (Artemiidae). *Pharmacology*.

[63] Oyetayo V. O., Nieto-Camacho A., Rodriguez B. E., Jimenez M. (2012). Assessment of Anti-Inflammatory, Lipid Peroxidation and Acute Toxicity of Extracts Obtained From Wild Higher Basidiomycetes Mushrooms Collected From Akure (Southwest Nigeria). *International Journal of Medicinal Mushrooms*.

[64] Kidukuli A. W., Mbwambo Z. H., Malebo H. M., Mgina C. A., Mihale M. J. (2010). *In Vivo* Antiviral Activity, Protease Inhibition and Brine Shrimp Lethality of Selected Tanzanian Wild Edible Mushrooms. *Journal of Applied Biosciences*.

[65] Ugbogu E. A., Emmanuel O., Ude V. C., Ijioma S. N., Ugbogu O. C., Akubugwo E. I. (2020). Nutritional Composition and Toxicity Profile of *Cantharellus* Species (Purple Mushroom) in Rats. *Scientific African*.

[66] Phan C. W., David P., Naidu M., Wong K. H., Sabaratnam V. (2013). Neurite Outgrowth Stimulatory Effects of Culinary-Medicinal Mushrooms and Their Toxicity Assessment Using Differentiating Neuro-2a and Embryonic Fibroblast BALB/3T3. *BMC Complementary and Alternative Medicine*.

